# Heavy metal exposure and nasal *Staphylococcus aureus* colonization: analysis of the National Health and Nutrition Examination Survey (NHANES)

**DOI:** 10.1186/s12940-017-0349-7

**Published:** 2018-01-05

**Authors:** Shoshannah Eggers, Nasia Safdar, Kristen MC Malecki

**Affiliations:** 1 Department of Population Health Sciences, School of Medicine and Public Health, University of Wisconsin – Madison, Warf Office Bldg, 610 Walnut St #707, Madison, WI 53726 USA; 2 Department of Medicine, School of Medicine and Public Health, University of Wisconsin – Madison, 750 Highland Ave, Madison, WI 53726 USA; 3 William S. Middleton Veterans Affairs Medical Center, 2500 Overlook Terrace, Madison, WI 53705 USA

**Keywords:** MRSA, MSSA, NHANES, Heavy metals, Environmental epidemiology, Lead, Pb, Cd, Cadmium, Antibiotic resistance

## Abstract

**Background:**

Heavy metals including lead and cadmium can disrupt the immune system and the human microbiota. and are increasingly of concern with respect to the propogation of antibiotic-resistence. Infection by methicillin-resistant *Staphylococcus aureus* (MRSA) is a major cause of global morbidity and mortality. Heavy metal exposure may be associated with increased MRSA colonization and infection, and a decrease in methicillin-susceptible *Staphylococcus aureus* (MSSA) through co-selection mechanisms and natural selection of antibiotic resistance in the presence of heavy metals. This study examines the association between blood lead (Pb) and cadmium (Cd) level, and MRSA and MSSA nasal colonization.

**Methods:**

All data used for this analysis came from the 2001–2004 National Health and Nutrition Examination Survey (NHANES). The analytical sample consisted of 18,626 participants aged 1 year and older. Multivariate logistic regression, including adjustment for demographic and dietary factors, was used to analyze the association between blood Pb and Cd, and nasal colonization by MRSA and MSSA.

**Results:**

Prevalence of MRSA and MSSA carriage were 1.2%, and 29.3% respectively. MRSA was highest in women, individuals age 70 and older, who self-identified as black, had only a high school diploma, lived below 200% of the Federal Poverty Level, and had a history of smoking. While not significantly different from those colonized with MSSA, geometric mean blood Pb (1.74 μg/dL) and blood Cd (0.31 μg/L) were highest in those colonized with MRSA. Associations with MRSA colonization appeared to increase in a dose-dependent manner with increasing quartile of blood Pb level. Blood Cd level in the fourth quartile was also significantly associated with lower odds of MRSA colonization. Both metals were associated with lower odds of MSSA colonization.

**Conclusions:**

Both MRSA and MSSA results suggest that general population levels of blood Pb but not Cd are associated with differences in nasal carriage of *S. aureus*. While further research is needed, reduction in heavy metal exposures such as lead, concurrently with maintaining a healthy microbiota may be two modifiable options to consider in the fight against antibiotic-resistance.

## Background

Exposure to heavy metals, such as lead (Pb) and cadmium (Cd), has been shown to reduce immune function, and has previously been associated with increased prevalence of infection [1–5]. While studies on the immunotoxicology of Cd have shown mixed results across varying conditions of exposure and immunological outcomes, cell-mediated immunity has consistently been depressed upon exposure to Cd [4]. Similarly, even low levels of Pb exposure have been shown to affect almost every aspect of immune function [6]. Some of the most prominent effects of Pb on the immune system are a shift toward Type 2 T helper Cell (Th2) response and increased Interleukin-4 secretion, reducing the Type 1 T helper Cell (Th1) response, and increasing autoimmune antibodies [6]. Beyond the typically reported adverse effects of lead exposure, such as neurological, renal, and developmental effects, and of cadmium, respiratory, cardiovascular, and renal effects, these changes in immune function caused by heavy metal exposure reduce the body’s ability to fight infection [7, 8].

Heavy metals are not only harmful to humans; they can often have toxic effects on bacteria, much like antibiotics. Moreover, metals are increasingly being incorporated into products for their antimicrobial properties. Considering the vast number of microbes that live on and within humans (the human microbiota), human exposure to heavy metals has the potential to influence our health not only by their toxicological effects on human cells and systems, but by altering our microbiota as well. Imbalance, or dysbiosis, of the microbiota have been linked to many adverse chronic health outcomes including infection [9–12]. Our microbiota play a large role in the development of the immune system, and continue to interact with the immune system to maintain homeostasis throughout our lives [13–15]. Beneficial bacteria within the microbiota produce cytokines, short and long chain fatty acids, and other signaling molecules that affect mucus production, epithelial barriers, and increase Th1 response [16, 17]. Heavy metal exposure could thereby reduce immune function indirectly through the microbiota, as well as through direct effects to the immune system, both increasing risk of infection. Not only is the risk for increased infection a concern, increasing antibiotic resistance of bacteria compound the potential health impact of these exposures.

Although heavy metals can be toxic to microbes, metal resistance, similar to antibiotic resistance, has been well documented across many different bacteria for many different metals [18–21]. Often, metal resistance and antibiotic resistance are present in the same bacteria [22–24]. There are multiple potential mechanisms for the co-selection of metal and antibiotic resistance genes within bacteria. Microbes can have two separate genes that code for metal and antibiotic resistance, which can either be physically linked within a genetic unit, like a plasmid, or can be transcriptionally linked, with one stimulus initiating transcription of both genes [22]. Alternatively, bacteria may have one gene that produces a protein set conferring resistance to both metals and antibiotics via the same mechanism [22]. In any of these cases, exposure to metal would not only cause bacteria to select for metal resistance, but antibiotic resistance as well.

Heavy metals such as Pb and Cd have been shown to co-select for metal and antibiotics in many different environmental settings, including groundwater, drinking water and wastewater [23–25]. Co-selection occurs through multiple mechanisms including co-resistence. Bacterial resistance to heavy metals and antibiotics are often associated as the genes that encode resistance can be physically or transcriptionally linked, or one gene can confer resistance to both antibiotics and metals by the same mechanism. Beyond studies of co-election in environmental media, studies on *Staphylococcus* isolates from animals and humans have shown associations between Pb and Cd resistance and antibiotic resistance [26, 27]. For example, fecal bacterial isolates from leghorn chickens that were fed Pb at levels that did not cause other morbidity or mortality, showed significantly elevated levels of antibiotic resistance compared to controls, and demonstrated a dose response relationship within different levels of Pb in the diet [28]. However, to our knowledge, few if any studies have examined the relationship between Pb or Cd exposure and selection for antibiotic resistance in humans.

Methicillin-resistant *Staphylococcus aureus* (MRSA) is a serious cause of bacteremia, resulting in 100,000 serious infections and 20,000 deaths in the US annually [29]. Although MRSA is commonly thought of as a health-care-acquired infection, it can be transmitted through the community as well, particularly in settings with a large amount of person to person contact [29, 30]. Those asymptomatically colonized by MRSA are at increased risk of infection compared to those who carry methicillin-susceptible *Staphylococcus aureus* (MSSA), and those who carry neither [31–33]. Antibiotic resistance and virulence factors can be easily transferred between bacteria, thus colonization by either MRSA or MSSA increases risk of infection. Prevention of MRSA colonization and infection is critical as effective treatment options are becoming increasingly sparse [34].

Reduction in heavy metal exposure concurrent with maintaining a healthy microbiota may be two modifiable options to consider in the fight against antibiotic-resistance. The mechanism by which heavy metal exposures such as ingestion of Pb and Cd could lead to antibiotic resistant infections and MRSA colonization is multifaceted. Presence of toxic metals in the digestive and circulatory system can alter the microbiome by reducing the abundance of metal susceptible bacteria, that are also likely antibiotic susceptible [35]. A healthy microbiome can help prevent antibiotic resistant infections by improving immune function and via competitive inhibition, in which commensal (non-disease causing, symbiotic) bacteria outcompete pathogens for finite resources like nutrients and mucosal binding sites. Reducing the number of commensal metal and antibiotic susceptible bacteria within the microbiome both reduces the capacity for competitive inhibition, and increases the prevalence of metal and antibiotic resistance within the remaining bacteria. Ultimately, Pb and Cd’s capacity to reduce competitive inhibition, select for metal and antibiotic resistance, either in vivo or in the environment, and inhibit immune function, make it highly plausible that Pb and Cd exposure in humans would be associated with colonization and subsequent infection by MRSA and other antibiotic resistant organisms.

Due to the paucity of human data available to study these potential mechanisms, this study aimed to examine human blood Pb and Cd levels and their associations with concurrent MRSA and MSSA nasal colonization among a general population based sample of United States residents. We hypothesized that circulating levels of Pb and Cd in blood would both be associated with an increase in MRSA colonization by co-selecting for metal and antibiotic resistance and a decrease in MSSA colonization by reducing the abundance of metal and antibiotic susceptible bacteria.

## Methods

### Data source

Data were abstracted from the 2001–2004 National Health and Nutrition Examination Survey (NHANES). NHANES is a population based survey including a wide range of determinants of health and health outcomes, incorporating a nationally representative sample of the U.S. population using a complex sampling framework and survey design [36, 37]. Data collection includes a household interview followed by a physical examination in which blood samples and nasal swabs were collected, along with additional bio-specimens and physical measurements (e.g. height, weight, blood pressure). Screening for MRSA and MSSA colonization occurred only during the 2001–2004 waves of the survey. Questionnaires used for data collection, and the publicly available data sets can be found on the NHANES website (https://www.cdc.gov/nchs/nhanes/nhanes_questionnaires.htm). The analytical sample for this study was 18,626 aged 1 year and older. This study was deemed exempt from review by the University of Wisconsin Institutional Review Board as it uses a publicly available, de-identified data set.

### Exposure measurement

Exposure to Pb and Cd were measured in whole blood samples collected during the physical examination portion of the survey. Blood lead levels of both elements were simultaneously analyzed in the laboratory using atomic absorption spectrophotometry [38] for years 2001–2002 and with inductively coupled plasma mass spectrometry in years 2003–2004 [39]. The lower limit of detection (LOD) was 0.30 μg/dL for Pb in 2001–2004, 0.30 μg/dL for Cd in 2001–2002, and 0.20 μg/dL for Cd in 2003–2004. Any results that were below the LOD were replaced by LOD/√2. Population distribution in whole blood concentrations of Pb and Cd were analyzed using geometric means, and quartiles.

### Outcome measurement

Colonization by *S. aureus* was tested using nasal swabs from eligible participants aged 1 year and older during the physical examination portion of the survey. Swabs were analyzed for the presence of *S. aureus* using standard culture based procedures [38, 39]. Identified *S. aureus* isolates were then tested for resistance to methicillin by disk diffusion. Participants that tested positive for *S. aureus* and negative for MRSA were considered positive for MSSA. Participants that tested negative for *S. aureus* were considered negative for both MRSA and MSSA. No data was collected on clinical infection status.

### Confounder measurement

Confounders included in the multivariate analysis for both Pb and Cd models were socio-demographics including gender, age, income, race and education, all of which were self-reported. Gender was modeled as a dichotomous variable, while age and income were both continuous. Age was categorized in 20-year increments, and income was categorized as above or below 200% of the Federal Poverty Level (FPL) for comparison in the descriptive statistics table. Race was included in the analysis of Pb because it has previously been associated with the outcome and exposure [40, 41]. Race was categorized as follows: non-Hispanic white, Mexican, other Hispanic, non-Hispanic black, and other. These categories are predefined in the data set and have been used in previous investigations of *S. aureus* and of blood Pb [42–44]. Education was categorized into three groups: those with less than a high school diploma, those with only a high school diploma, and those with a high school diploma and at least some college. Smoking status was included in the analysis of both Pb and Cd, as it is a source of exposure to both metals and may be associated with MRSA [45–47]. Smoking status was self-reported and categorized into current, former, and never smokers. Smoking data was not collected for children under the age of 12. They are assumed to be never smokers.

Diet data were also included in final adjusted models, and different micronutrients were considered separately for Pb and Cd models. A 24-h dietary recall was collected at least once for all eligible participants in 2001–2004, and at least twice for a subsample of participants in 2003–2004. The data used in this analysis was from the first dietary recall only. Total grams of Iron, Calcium, and Vitamin C were included in the analysis of Pb as they have previously been shown to affect Pb absorption, [48, 49] and are likely associated with consumption of a dietary factors that could affect *S. aureus* colonization and infection, including diets with increased fiber. Dietary factors associated with Cd exposure and potentially *S. aureus* carriage are consumption of green leafy vegetables [50]. Data on individual food consumption is categorized by United States Department of Agriculture (USDA) food codes [51]. These codes were used to identify all fruits and vegetables eaten and calculate total grams of fruit and vegetable consumption. All dietary variables were modeled as continuous.

### Statistical analysis

Statistical analysis was performed using SAS v.9.4 (Carry, NC). SAS survey procedures were used with 4 year laboratory weights calculated as recommended in NHANES analytic guidelines to account for probability sampling design and clustering [36]. Descriptive statistics including unadjusted frequencies of socio-demographic factors, means of dietary intake, and geometric means of blood Pb and Cd level were calculated. Multivariate logistic regression was used to evaluate the associations between blood Pb and Cd level and MRSA and MSSA colonization. Models were built using a priori knowledge of associations to avoid use of covariates that are spuriously correlated, and that were identified as confounders using a direct acyclic graph (DAG). To achieve more parsimonious models, backwards selection was used to remove variables that were not significant at the *p* ≤ 0.02 level. A series of three logistic regression models were run to examine the association between increasing quartiles of whole blood Pb or Cd as the exposure, and either MRSA and MSSA as the outcome. Model 1 was unadjusted for both Pb and Cd. Model 2 adjusted for significant socio-demographics (age, gender, race, and income for Pb, and age, gender, and income for Cd), and Model 3 added smoking and dietary factors (Iron, Calcium, and Vitamin C to the Pb model, and fruit and vegetable consumption to the Cd model) to Model 2. Results are considered statistically significant with a *p*-value ≤0.05, or a confidence interval that does not cross 1.00 for odds ratio estimates.

## Results

Population levels of exposure based on quartiles (Q) of blood Pb level were: Q1 = 0.0–0.90 μg/dL, Q2 = 0.91–1.40 μg/dL, Q3 = 1.41–2.30 μg/dL, Q4 = 2.31–68.9 μg/dL. Similarly, quartiles of blood Cd level were: Q1 = 0.0–0.20 μg/L, Q2 = 0.21–0.30 μg/L, Q3 = 0.31–0.50 μg/L, Q4 = 0.51–7.4 μg/L. Descriptive statistics in Table 1 show that the prevalence of MRSA was 1.2%, and MSSA was 29.3%. MRSA carriage is highest in those age 70 and above, females, Non-Hispanic Blacks and Non-Hispanic Whites, those below 200% FPL, and those who have ever smoked cigarettes. Prevalence of MRSA was very similar across levels of education, but slightly higher in those with a high school diploma only. Carriage of MSSA was highest in those age 29 and younger, males, Non-Mexican Hispanics, those with less than a high school diploma, those above 200% FPL, and those who have never smoked cigarettes. Geometric mean blood Pb and Cd are both highest in those with MRSA, and lowest in those with MSSA. Mean Iron, Vitamin C, and fruit and vegetable consumption are highest in those with MRSA, and mean Calcium consumption is highest in those with MSSA.

**Table 1:** Prevalence of demographic factors by *Staphylococcus aureus* colonization status

	None	MSSA	MRSA
Demographics	n (%)	n (%)	n (%)
Total	13,220 (69.5)	5198 (29.3)	208 (1.2)
Age ***			
1–17	5419 (63.8)	2521 (35.3)	72 (0.9)
18–29	2063 (69.4)	819 (29.7)	19 (0.9)
30–49	2336 (70.8)	855 (28.4)	29 (0.8)
50–69	1919 (71.8)	631 (26.2)	43 (1.9)
70 +	1483 (76.9)	372 (21.0)	45 (2.1)
Sex ***			
Male	6211 (66.2)	2794 (32.9)	93 (1.0)
Female	7009 (72.7)	2404 (25.9)	115 (1.4)
Race ***			
White	5293 (68.4)	2254 (30.3)	102 (1.3)
Mexican	3334 (71.2)	1287 (28.1)	33 (0.7)
Other Hispanic	471 (64.1)	265 (35.7)	2 (0.2)
Black	3559 (75.3)	1196 (23.3)	64 (1.4)
Other	563 (73.3)	196 (25.8)	7 (0.9)
Education **			
< High School Diploma	6063 (66.0)	2753 (32.8)	93 (1.2)
High School Diploma	1971 (72.2)	695 (26.5)	30 (1.3)
> High School Diploma	3317 (70.0)	1239 (28.9)	54 (1.1)
Income *			
< 200% FPL	6475 (69.9)	2491 (28.6)	121 (1.5)
≥ 200% FPL	5829 (69.6)	2344 (29.5)	72 (0.9)
Smoking ***			
Current Smoker	2105 (75.1)	664 (23.8)	31 (1.1)
Former Smoker	2217 (70.4)	771 (27.5)	57 (2.1)
Never Smoker	8877 (67.4)	3781 (31.7)	120 (0.9)
Metals	GM (95% CI)	GM (95% CI)	GM (95% CI)
Blood Lead *ug/dL*	1.50 (1.48, 1.52)	1.43 (1.40, 1.45)	1.74 (1.58, 1.93)
Blood Cadmium *ug/L*	0.30 (0.30, 0.31)	0.27 (0.26, 0.28)	0.31 (0.28, 0.35)
Diet	Mean (95% CI)	Mean (95% CI)	Mean (95% CI)
Iron *g*	14.89 (14.73, 15.05)	15.52 (15.25, 15.79)	16.13 (14.71, 17.55)
Calcium *g*	875.25 (864.87, 885.64)	922.35 (905.00, 939.70)	876.08 (792.21, 959.95)
Vitamin C *g*	97.95 (96.4, 99.86)	94.22 (91.44, 97.00)	112.78 (94.16, 131.40)
Fruits and Vegetables *g*	372.23 (363.41, 381.04)	340.60 (327.31, 353.89)	410.52 (338.23, 482.81)

*P ≤ 0.05;**P ≤ 0.005; ***P ≤ 0.0005. Data from NHANES 2001–2004, *n* = 18,626. Percentages are adjusted using survey weights to be representative of the United States population. Abbreviations: NHANES – National Health and Nutrition Examination Survey; MSSA – Methicillin-susceptible *Staphylococcus aureus*; MRSA – Methicillin-resistant *Staphylococcus aureus*; FPL – Federal Poverty Level; GM – Geometric mean; CI – Confidence interval

Logistic regression results for MRSA (Table 2) show that the fourth quartile of blood Pb level is associated with significantly increased odds of MRSA carriage in the unadjusted model. Although the effect size is similar in the fully adjusted model, the confidence interval crosses 1.00. Notably, there is a consistent dose response relationship with increased odds of MRSA colonization with increased quartile of blood Pb, across all models of Pb (p for trend = 0.0258). The unadjusted Cd model suggests increased odds of MRSA colonization with increased blood Cd level, however, after adjustment for diet, smoking and socio-demographics, the association between Cd and MRSA appears to be protective, with those in the second quartile having significantly reduced odds of MRSA carriage. Analysis examining MSSA as the outcome (Table 3) demonstrate marginally decreased odds of MSSA colonization for those in the highest blood Pb group (p for trend = 0.0044). Similarly, blood Cd in the fourth quartile is significantly associated with decreased odds of MSSA carriage, and shows a dose response effect across all models (p for trend = <.0001).

**Table 2:** Results of logistic regression with MRSA colonization as the outcome

	Q1	Q2	Q3	Q4
	OR	OR (95% CI)	OR (95% CI)	OR (95% CI)
Pb*				
Model 1^a^	1.00	1.44 (0.82, 2.55)	1.59 (0.91, 2.78)	**1.82** (1.01, 3.29)
Model 2^b^	1.00	1.27 (0.71, 2.26)	1.36 (0.65, 2.88)	1.52 (0.66, 3.51)
Model 3^c^	1.00	1.52 (0.83, 2.76)	1.56 (0.75, 3.24)	1.82 (0.81, 4.10)
Cd				
Model 1^a^	1.00	0.63 (0.38, 1.03)	1.21 (0.71, 2.07)	1.26 (0.85, 1.86)
Model 2^d^	1.00	**0.50** (0.29, 0.86)	0.74 (0.45, 1.23)	0.82 (0.50, 1.35)
Model 3^e^	1.00	**0.41** (0.20, 0.83)	0.60 (0.34, 1.08)	0.60 (0.36, 1.03)

*P for trend ≤0.05. a) Unadjusted; b) Adjusted for age, gender, race, and income; c) Adjusted for age, gender, race, income, smoking, iron, calcium, and Vitamin C; d) Adjusted for age, gender, income, and smoking; e) Adjusted for age, gender, income, smoking, and fruit and vegetable consumption. Data from NHANES 2001–2004, n = 18,626. Percentages are adjusted using survey weights to be representative of the United States population. Bold text indicates that the 95% CI does not cross 1.00, and the finding is considered significant. Abbreviations: MRSA – Methicillin-resistant *Staphylococcus aureus*; Q – Quartile; OR – Odds ratio; CI – Confidence interval; Pb – Lead; Cd – Cadmium

**Table 3:** Results of logistic regression with MSSA colonization as the outcome

	Q1	Q2	Q3	Q4
	OR	OR (95% CI)	OR (95% CI)	OR (95% CI)
Pb**				
Model 1^a^	1.00	1.01 (0.89, 1.14)	0.96 (0.84, 1.11)	**0.79** (0.67, 0.92)
Model 2^b^	1.00	1.05 (0.93, 1.18)	1.05 (0.91, 1.21)	**0.84** (0.69, 1.00)
Model 3^c^	1.00	1.07 (0.95, 1.21)	1.10 (0.94, 1.28)	0.91 (0.76, 1.09)
Cd***				
Model 1^a^	1.00	**0.87** (0.76, 1.00)	**0.74** (0.64, 0.85)	**0.57** (0.50, 0.66)
Model 2^d^	1.00	0.96 (0.82, 1.11)	0.88 (0.75, 1.03)	**0.67** (0.58, 0.78)
Model 3^e^	1.00	1.16 (0.95, 1.42)	0.86 (0.66, 1.20)	**0.77** (0.60, 0.99)

**P for trend ≤0.005. ***P for trend ≤0.0001. a) Unadjusted; b) Adjusted for age, gender, race, and income; c) Adjusted for age, gender, race, income, smoking, iron, calcium, and Vitamin C; d) Adjusted for age, gender, income, and smoking; e) Adjusted for age, gender, income, smoking, and fruit and vegetable consumption. Data from NHANES 2001–2004, n = 18,626. Percentages are adjusted using survey weights to be representative of the United States population. Bold text indicates that the 95% CI does not cross 1.00, and the finding is considered significant. Abbreviations: MRSA – Methicillin-resistant *Staphylococcus aureus*; Q – Quartile; OR – Odds ratio; CI – Confidence interval; Pb – Lead; Cd – Cadmium

## Discussion

In this analysis of exposure to Pb and Cd measured as circulating levels of each in whole blood from a large scale, nationally representative sample of US residents, blood level of either metal was associated with differences in nasal carriage of *S. aureus*. As hypothesized, blood Pb level was also associated with increased odds of MRSA colonization, and decreased odds of MSSA colonization in minimally adjusted models controlling for age, gender, race and income. At the same time, increasing blood Cd level was associated with decreasing odds of both MRSA and MSSA colonization. Results of this first investigation support the idea that individuals with highest general population levels of heavy metals in blood, may be more susceptible to antibiotic resistence, and that associations likely vary by microbiotic diversity, type of metal and their toxicological properties including metabolism, and that the complex relationship between antimicrobial resistance and heavy metal exposure in humans warrants further exploration.

**Fig. 1:**
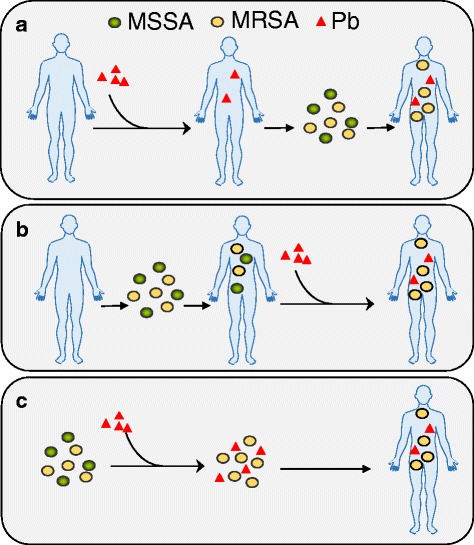
Diagram of the Natural Histories of Pb and MRSA Exposure. The natural history of Pb exposure and selection for antibiotic resistance in *Staphylococcus aureus* that colonize individuals in this study population could work in several ways. **a** The individual is exposed to Pb first, and is then exposed to MRSA and MSSA. The Pb prevents colonization by MSSA, but not MRSA. **b** The individual is colonized by MRSA and/or MSSA first, and is then exposed to Pb. The Pb selects for antibiotic resistance by killing MSSA and leaving MRSA behind. **c** MRSA and/or MSSA is exposed to Pb in the environment. The Pb exposure selects for antibiotic resistance in the environment. The individual is then exposed to both MRSA and Pb from the same source. Abbreviations: MRSA – Methicillin-resistant *Staphylococcus aureus*; MSSA – Methicillin susceptible *Staphylococcus aureus*; Pb – Lead

While, to our knowledge, few if any other studies of MRSA and heavy metal exposures in humans has been conducted to date, our findings of a dose response effect between Pb and MRSA with highest quartiles of exposure showing the greatest associations are consistent with work done in vitro and animal models [26, 28, 52]. Nisanian et al. was able to prospectively show antibiotic resistance as a result of Pb ingestion in chickens [28]. However, the association between Pb and MRSA in our study has multiple potential natural histories (Fig. 1) and further prospective work is needed to establish causality among human populations. A possible situation is that individuals were exposed to Pb prior to exposure to *S. aureus*, in which case the Pb exposure would reduce healthy gut microbiota, and fail to protect against the Pb- and antibiotic-resistant strains, leading to MRSA colonization, assuming the MRSA was also Pb resistant (Fig. 1a). At the same time, in this situation, assuming the antibiotic susceptible *S. aureus* bacteria (MSSA) is susceptible to Pb, the prior Pb exposure would likely protect against MSSA colonization. Alternatively, participants may be colonized by *S. aureus* before Pb exposure occurs, in which case that exposure selects for increased anti-microbial resistance and subsequent MRSA colonization in vivo (Fig. 1b). Otherwise, *S. aureus* could be exposed to Pb in the environment, co-selecting for resistance there, and the participant would then be exposed to both Pb and MRSA from the same source (Fig. 1c). It is not possible based on our findings to determine which one of these potential natural histories is most likely, however, this study is a first step to confirm that environmental exposures to heavy metals such as Pb may alter bacterial colonization and thus also have a larger influence on human microbial composition over time.

While the protective effect of Cd against MRSA did not support our hypothesis, it is not completely inconsistent with previous findings. While some studies have found that exposure to Cd led to antibiotic resistance in various bacteria, [25, 27] a study by Calomiris, et al., did not find an association between Cd and multiple antibiotic resistance in *Staphylococcus* isolated from drinking water [24]. Furthermore, while Cd and antibiotic resistance are often plasma linked, complex relationships between the host cells and plasmids can exist regarding resistance to metals [53]. It is likely that the strains of MRSA found in this population are not resistant to the toxic effects of Cd, explaining the protective effect found against both MRSA and MSSA. In addition, Cd exposure is linked with covariates contributing to increased host-resistance factors such as higher consumption of green-leafy vegetables, which may counter-act and confound associations between Cd exposure and immune function.

Infectious diseases, including MRSA, affect a disproportionately high number of people who are economically and socially disadvantaged at the individual, household, and neighborhood level [30, 54–58]. The association between Pb and MRSA colonization may be an important key to understanding the relationship between socioeconomic status (SES) and increased risk of infection. Because Pb exposure is often associated with low income, it is possible that PB exposure explains part of the biological mechanism linking SES and MRSA, as well as other infectious diseases. Exploration of this relationship via a formal mediation analysis using prospectively collected longitudinal data may be warranted.

These results considered as a whole suggest that both Pb and Cd are associated with differences in *S. aureus* carriage. These differences are likely apparent in other bacteria within the human microbiota as well. The human microbiome is becoming increasingly recognized as an important determinant of health throughout the life course, and exposure to xenobiotics, including metals, can influence the balance of our microbial ecosystems [35]. To date, investigation into the association between Pb and Cd and the microbiome have been done predominantly in animal models [59–66]. One study has examined the link between Pb and the human gut microbiota in children, and found a significant difference in abundance of Succinivibrionaceae and Gammaproteobacteria with elevated blood Pb [67]. Although their study is a good first step, and our findings confirm the association between Pb and the human nasal microbiota, further investigation with larger and more diverse samples are needed.

While these findings are an important step in the investigation between heavy metal exposure and antibiotic resistance in humans, this study is not without limitations. An important consideration is that these data are cross-sectional, thus temporal precedence, and therefore causation, cannot be asserted. Measurement of *S. aureus* colonization using only nasal swabs limits the level of detection, [68, 69] and makes the connection with Pb and Cd exposure slightly more tenuous as their exposure mechanisms are primarily through the gastrointestinal tract. However, individuals colonized by *S. aureus* are often colonized at multiple body sites, [68, 70, 71] thus exposure to Pb and Cd could have occurred at other body sites, or in the environment. No data were collected on clinical infections by *S. aureus*, however, colonization greatly increases risk of infection [32]. These data were also collected over a decade ago, and population levels of Pb and Cd exposure, and MSSA and MRSA carriage may have changed in that time. To our knowledge, no other data measuring these exposures and outcomes exists on a nationally representative scale for years after 2004. While these data may not be representative of current population estimates, they are still useful in examining biological associations that may exist. Residual confounding may be a concern, therefore, future examination of these associations would benefit from having more thorough colonization screening including multiple body sites, and less reliance on self-reported data.

Metal half-life in blood is between 28 and 36 days for Pb, [72] and between 75 and 128 days for Cd [73]. Measuring exposure to Pb and Cd in whole blood may not be the best measurement of exposure by bacteria in the microbiota. Because microbial communities are not only affected by xenobiotic exposure, but can also influence the metabolism and level of absorption of those xenobiotics into the bloodstream, [35] part of the association found in this study may be due to reverse causality. For instance, if an individual in this study is colonized by MRSA in the gut and the nose, it could be that the MRSA present in the gut is Pb-resistant by efflux of the Pb from the cell. Colonization by such bacteria would therefor increase the level of Pb available for absorption by the human, as it is not being absorbed by these Pb-resistant bacteria. That higher availability of Pb could then be reflected in the blood Pb measurement. In such a case, the blood Pb would be elevated because of the presence of MRSA, as opposed to increased Pb exposure increasing risk of MRSA colonization. Furthermore, it is not clear whether acute or chronic exposures have a greater influence on the association with *S. aureus*, as source and length of *S. aureus* colonization cannot be determined. Future studies are needed to determine the best measurement matrix to measure human microbiota exposure to xenobiotics.

The results of this analysis could be expanded into many different future investigations, including the mediation and microbiome analyses previously mentioned. Because not all metal and antibiotic resistance mechanisms are alike, the association between Pb and Cd in human populations could be investigated with other antibiotic resistant bacteria as the outcome of interests. Likewise, the investigation between other heavy metals and MRSA, as well as other antibiotic resistant bacteria could be further developed. Observational studies within a clinical setting, examining Pb exposure and its association with symptomatic MRSA infections and their clinical outcomes is another potential line of inquiry.

## Conclusions

To our knowledge, this analysis is among the first to investigate the association between Pb and Cd exposure and colonization by MRSA in a human population. Results suggest that current levels of blood lead in the population, particularly among those in the highest quartile of estimated exposures, is associated with differences in nasal carriage of antibiotic-resistant *S. aureus*. The protective effect of Cd against both antibiotic resistant *S.auras* (MRSA) and antibiotic susceptible (MSSA) suggests either MRSA may not be resistant to the toxic effects of Cd, or that this relationship may be affected by immunomodulation. Reducing Pb exposure could become a useful strategy for preventing MRSA colonization and infection.

## Abbreviations

Cd: Cadmium
CI: Confidence Interval
DAG: Directed Acyclic Graph
FPL: Federal Poverty Level
LOD: Limit of Detection
MRSA: Methicillin-resistant *Staphylococcus aureus*
MSSA: Methicillin-susceptible *Staphylococcus aureus*
NHANES: National Health and Nutrition Examination Survey
OR: Odds Ratio
Pb: Lead
Q: Quartiles
SES: Socioeconomic Status
Th1: Type 1 T helper Cell
Th2: Type 2 T helper Cell
USDA: United States Department of Agriculture

## References

[1] Ninkov M, Popov Aleksandrov A, Demenesku J, Mirkov I, Mileusnic D, Petrovic A (2015). Toxicity of oral cadmium intake: impact on gut immunity. Toxicol Lett.

[2] Zhang Q, Huang Y, Zhang K, Huang Y, Yan Y, Wang F (2016). Cadmium-induced immune abnormality is a key pathogenic event in human and rat models of preeclampsia. Environ. Pollut. Barking Essex 1987.

[3] Lafuente A, González-Carracedo A, Esquifino AI (2004). Differential effects of cadmium on blood lymphocyte subsets. Biometals.

[4] Descotes J. Immunotoxicology of cadmium. IARC Sci Publ. 1992:385–90. 1303965

[5] Krueger WS, Wade TJ. Elevated blood lead and cadmium levels associated with chronic infections among non-smokers in a cross-sectional analysis of NHANES data. Environ. Health [Internet]. 2016 [cited 2016 Sep 22];15. Available from: http://www.ncbi.nlm.nih.gov/pmc/articles/PMC4750187/10.1186/s12940-016-0113-4 PMC4750187 26864738

[6] Dietert RR, Piepenbrink MS (2006). Lead and immune function. Crit Rev Toxicol.

[7] Abadin H, Ashizawa A, Stevens Y-W, Llados F, Diamond G (2007). Sage G, et al.

[8] Bernhoft RA. Cadmium Toxicity and Treatment. Sci. World J. [Internet]. 2013;2013. Available from: https://www.ncbi.nlm.nih.gov/pmc/articles/PMC3686085/10.1155/2013/394652 PMC3686085 23844395

[9] Sekirov I, Russell SL, Antunes LCM, Finlay BB (2010). Gut microbiota in health and disease. Physiol Rev.

[10] Bull MJ, Plummer NT (2014). Part 1: the human gut microbiome in health and disease. Integr. Med. Clin J.

[11] Keesing F, Belden LK, Daszak P, Dobson A, Harvell CD, Holt RD (2010). Impacts of biodiversity on the emergence and transmission of infectious diseases. Nature.

[12] Madan JC, Salari RC, Saxena D, Davidson L, O’Toole GA, Moore JH (2012). Gut microbial colonisation in premature neonates predicts neonatal sepsis. Arch Dis Child - Fetal Neonatal Ed.

[13] Hooper LV, Littman DR, Macpherson AJ (2012). Interactions between the microbiota and the immune system. Science.

[14] Lee YK, Mazmanian SK (2010). Has the microbiota played a critical role in the evolution of the adaptive immune system?. Science.

[15] Round JL, Mazmanian SK (2009). The gut microbiota shapes intestinal immune responses during health and disease. Nat Rev Immunol.

[16] den Besten G, van Eunen K, Groen AK, Venema K, Reijngoud D-J, Bakker BM (2013). The role of short-chain fatty acids in the interplay between diet, gut microbiota, and host energy metabolism. J Lipid Res.

[17] Cénit MC, Matzaraki V, Tigchelaar EF, Zhernakova A (1842). Rapidly expanding knowledge on the role of the gut microbiome in health and disease. Biochim Biophys Acta (BBA) - Mol Basis Dis.

[18] Nies DH (1999). Microbial heavy-metal resistance. Appl Microbiol Biotechnol.

[19] Nies DH (2003). Efflux-mediated heavy metal resistance in prokaryotes. FEMS Microbiol Rev.

[20] Naik MM, Dubey SK (2013). Lead resistant bacteria: lead resistance mechanisms, their applications in lead bioremediation and biomonitoring. Ecotoxicol Environ Saf.

[21] Banerjee PC. Genetics of metal resistance in acidophilic prokaryotes of acidic mine environments. IJEB Vol4201 January 2004 [Internet]. 2004 [cited 2017 Apr 25]; Available from: http://nopr.niscair.res.in/handle/123456789/23315 15274476

[22] Baker-Austin C, Wright MS, Stepanauskas R, McArthur JV (2006). Co-selection of antibiotic and metal resistance. Trends Microbiol.

[23] Aktan Y, Tan S, Icgen B (2013). Characterization of lead-resistant river isolate enterococcus faecalis and assessment of its multiple metal and antibiotic resistance. Environ Monit Assess.

[24] Calomiris JJ, Armstrong JL, Seidler RJ (1984). Association of metal tolerance with multiple antibiotic resistance of bacteria isolated from drinking water. Appl Environ Microbiol.

[25] Patra S, Das TK, Avila C, Cabello V, Castillo F, Sarkar D (2012). Cadmium tolerance and antibiotic resistance in Escherichia Coli isolated from waste stabilization ponds. Indian J Exp Biol.

[26] Ug A, Ceylan Ö (2003). Occurrence of resistance to antibiotics, metals, and plasmids in clinical strains of staphylococcus spp. Arch Med Res.

[27] Nair R, Thapaliya D, Su Y, Smith TC (2014). Resistance to zinc and cadmium in Staphylococcus Aureus of human and animal origin. Infect Control Hosp Epidemiol.

[28] Nisanian M, Holladay SD, Karpuzoglu E, Kerr RP, Williams SM, Stabler L (2014). Exposure of juvenile leghorn chickens to lead acetate enhances antibiotic resistance in enteric bacterial flora. Poult Sci.

[29] Klevens R, Morrison MA, Nadle J (2007). INvasive methicillin-resistant staphylococcus aureus infections in the united states. JAMA.

[30] Tosas Auguet O, Betley JR, Stabler RA, Patel A, Ioannou A, Marbach H (2016). Evidence for community transmission of community-associated but not health-care-associated methicillin-resistant staphylococcus aureus strains linked to social and material deprivation: spatial analysis of cross-sectional data. PLoS Med.

[31] Davis KA, Stewart JJ, Crouch HK, Florez CE, Hospenthal DR (2004). Methicillin-resistant Staphylococcus Aureus (MRSA) nares colonization at hospital admission and its effect on subsequent MRSA infection. Clin Infect Dis.

[32] Safdar N, Bradley EA (2008). The risk of infection after nasal colonization with staphylococcus aureus. Am J Med.

[33] Coello R, Glynn JR, Gaspar C, Picazo JJ, Fereres J (1997). Risk factors for developing clinical infection with methicillin-resistant Staphylococcus Aureus (MRSA) amongst hospital patients initially only colonized with MRSA. J Hosp Infect.

[34] Center for Disease Control and Prevention. Antibiotic Resistance Threats in the United States, 2013. U.S. Department of Health and Human Services; 2013 Apr.

[35] Claus SP, Guillou H, Ellero-Simatos S (2016). The gut microbiota: a major player in the toxicity of environmental pollutants?. Npj Biofilms Microbiomes.

[36] Johnson CL, Paulose-Ram R, Ogden C e, Carroll MD, Kruszon-Moran D, Dohrmann SM, et al. National health and nutrition examination survey: analytic guidelines, 1999-2010. Vital Health Stat 2. 2013;1–24. 25090154

[37] NHANES - About the National Health and Nutrition Examination Survey [Internet]. 2017 [cited 2017 Oct 31]. Available from: https://www.cdc.gov/nchs/nhanes/about_nhanes.htm

[38] NHANES - 2001-2002 Lab Methods [Internet]. [cited 2017 Apr 25]. Available from: https://wwwn.cdc.gov/nchs/nhanes/continuousnhanes/labmethods.aspx?BeginYear=2001

[39] NHANES - 2003-2004 Lab Methods [Internet]. [cited 2017 Apr 25]. Available from: https://wwwn.cdc.gov/nchs/nhanes/continuousnhanes/labmethods.aspx?BeginYear=2003

[40] Mainous AG, Hueston WJ, Everett CJ, Diaz VA (2006). Nasal carriage of Staphylococcus Aureus and methicillin-resistant S aureus in the United States, 2001-2002. Ann Fam Med.

[41] Wells EM, Bonfield TL, Dearborn DG, Jackson LW (2014). The relationship of blood lead with immunoglobulin E, eosinophils, and asthma among children: NHANES 2005–2006. Int J Hyg Environ Health.

[42] NHANES - Questionnaires, Datasets, and Related Documentation [Internet]. [cited 2015 Nov 11]. Available from: http://www.cdc.gov/nchs/nhanes/nhanes_questionnaires.htm

[43] Davis MF, Peng RD, McCormack MC, Matsui EC. *Staphylococcus aureus* colonization is associated with wheeze and asthma among US children and young adults. J. Allergy Clin. Immunol. 2015;135:811–813.e5. 10.1016/j.jaci.2014.10.052 PMC4955790 25533526

[44] Buser MC, Ingber SZ, Raines N, Fowler DA, Scinicariello F (2016). Urinary and blood cadmium and lead and kidney function: NHANES 2007–2012. Int J Hyg Environ Health.

[45] Tellez-Plaza M, Navas-Acien A, Caldwell KL, Menke A, Muntner P, Guallar E (2012). Reduction in cadmium exposure in the United States population, 1988-2008: the contribution of declining smoking rates. Environ Health Perspect.

[46] Wooten DA, Winston LG (2013). Risk factors for methicillin-resistant Staphylococcus Aureus in patients with community-onset and hospital-onset pneumonia. Respir Med.

[47] Mortada WI, Sobh MA, El-Defrawy MM (2004). The exposure to cadmium, lead and mercury from smoking and its impact on renal integrity. Med Sci Monit Int Med J Exp Clin Res.

[48] Zentner LEA, Rondó PHC, Duran MC, Oliveira JM (2008). Relationships of blood lead to calcium, iron, and vitamin C intakes in Brazilian pregnant women. Clin Nutr.

[49] Jiao J, Lü G, Liu X, Zhu H, Zhang Y (2011). Reduction of blood lead levels in lead-exposed mice by dietary supplements and natural antioxidants. J Sci Food Agric.

[50] Garg VK, Yadav P, Mor S, Singh B, Pulhani V (2014). Heavy metals bioconcentration from soil to vegetables and assessment of health risk caused by their ingestion. Biol Trace Elem Res.

[51] Fndds download databases: USDA ARS [Internet]. [cited 2017 Oct 31]. Available from: https://www.ars.usda.gov/northeast-area/beltsville-md/beltsville-human-nutrition-research-center/food-surveys-research-group/docs/fndds-download-databases/.

[52] Nakahara H, Ishikawa T, Sarai Y, Kondo I (1977). Distribution of resistances to metals and antibiotics of staphylococcal strains in Japan. Zentralblatt Bakteriol. Parasitenkd Infekt Hyg Erste Abt Orig Reihe Med Mikrobiol Parasitol.

[53] Novick RP, Roth C (1968). Plasmid-linked resistance to inorganic salts in Staphylococcus Aureus. J Bacteriol.

[54] Semenza JC, Giesecke J (2008). Intervening to reduce inequalities in infections in Europe. Am J Public Health.

[55] Semenza JC, Suk JE, Tsolova S, Gillespie IA, Mook P, Little CL (2010). Social determinants of infectious diseases: a public health priority. Euro Surveill.

[56] Bagger JP, Zindrou D, Taylor KM (2004). Postoperative infection with meticillin-resistant Staphylococcus Aureus and socioeconomic background. Lancet.

[57] Farr AM, Marx MA, Weiss D, Nash D (2013). Association of neighborhood-level factors with hospitalization for community-associated methicillin-resistant Staphylococcus Aureus, new York City, 2006: a multilevel observational study. BMC Infect Dis.

[58] Davis MF, Peterson AE, Julian KG, Greene WH, Price LB, Nelson K (2013). Household risk factors for colonization with multidrug-resistant Staphylococcus Aureus isolates. PLoS One.

[59] Wu J, Wen XW, Faulk C, Boehnke K, Zhang H, Dolinoy DC (2016). Perinatal lead exposure alters gut microbiota composition and results in sex-specific bodyweight increases in adult mice. Toxicol Sci.

[60] Li Y, Liu K, Shen J, Liu Y (2016). Wheat bran intake can attenuate chronic cadmium toxicity in mice gut microbiota. Food Funct.

[61] Zhai Q, Narbad A, Chen W (2015). Dietary strategies for the treatment of cadmium and lead toxicity. Nutrients.

[62] Zhang S, Jin Y, Zeng Z, Liu Z, Fu Z (2015). Subchronic exposure of mice to cadmium perturbs their hepatic energy metabolism and gut microbiome. Chem Res Toxicol.

[63] Zhang W, Guo R, Yang Y, Ding J, Zhang Y (2016). Long-term effect of heavy-metal pollution on diversity of gastrointestinal microbial community of Bufo Raddei. Toxicol Lett.

[64] Breton J, Massart S, Vandamme P, De Brandt E, Pot B, Foligné B (2013). Ecotoxicology inside the gut: impact of heavy metals on the mouse microbiome. BMC. Pharmacol Toxicol.

[65] Ba Q, Li M, Chen P, Huang C, Duan X, Lu L (2017). Sex-dependent effects of cadmium exposure in early life on gut microbiota and fat accumulation in mice. Environ Health Perspect.

[66] Liu Y, Li Y, Liu K, Shen J (2014). Exposing to cadmium stress cause profound toxic effect on microbiota of the mice intestinal tract. PLoS One.

[67] Bisanz JE, Enos MK, Mwanga JR, Changalucha J, Burton JP, Gloor GB (2014). Randomized open-label pilot study of the influence of probiotics and the gut microbiome on toxic metal levels in Tanzanian pregnant women and school children. MBio.

[68] Girou E, Azar J, Wolkenstein P, Cizeau F, Brun-Buisson C, Roujeau J-C (2000). Comparison of systematic versus selective screening for methicillin-resistant <span class=“italic”>Staphylococcus Aureus</span> carriage in a high-risk dermatology Ward. Infect Control Amp Hosp Epidemiol.

[69] Datta P, Vasdeva HR, Chander J (2013). Optimization of multiple muco-cutaneous site sampling method for screening MRSA colonization in ICU. Indian J. Crit Care Med.

[70] Singh J, Johnson RC, Schlett CD, Elassal EM, Crawford KB, Mor D (2016). Multi-body-site microbiome and culture profiling of military trainees suffering from skin and soft tissue infections at fort Benning, Georgia. mSphere.

[71] Sim BLH, McBryde E, Street AC, Marshall C (2013). Multiple site surveillance cultures as a predictor of methicillin-resistant Staphylococcus Aureus infections. Infect Control Hosp Epidemiol.

[72] Sakai T (2000). Biomarkers of lead exposure. Ind Health.

[73] Järup L, Rogenfelt A, Elinder C-G, Nogawa K, Kjellström T (1983). Biological half-time of cadmium in the blood of workers after cessation of exposure. Scand. J. Work. Environ Health.

